# Magnetic Resonance Imaging Features Associated with a High and Low Expression of Tumor-Infiltrating Lymphocytes: A Stratified Analysis According to Molecular Subtypes

**DOI:** 10.3390/cancers15235672

**Published:** 2023-11-30

**Authors:** Jiejie Zhou, Yi Jin, Haiwei Miao, Shanshan Lu, Xinmiao Liu, Yun He, Huiru Liu, Youfan Zhao, Yang Zhang, Yan-Lin Liu, Zhifang Pan, Jeon-Hor Chen, Meihao Wang, Min-Ying Su

**Affiliations:** 1 Department of Radiology, First Affiliated Hospital of Wenzhou Medical University, Wenzhou 325000, China; zhoujiejie1984@163.com (J.Z.); carolmhw@126.com (H.M.); liuxinmiao199710@163.com (X.L.); hy15267755272@163.com (Y.H.); liuhuiru0203@126.com (H.L.); zhaoyoufan@wmu.edu.cn (Y.Z.); 2 Department of Radiological Sciences, University of California, Irvine, CA 92697, USA; yangz17@uci.edu (Y.Z.); yanlin0917@gmail.com (Y.-L.L.); jeonhc@uci.edu (J.-H.C.); 3 Department of Pathology, First Affiliated Hospital of Wenzhou Medical University, Wenzhou 325000, China; jinyi1029@126.com (Y.J.); 13958937000@163.com (S.L.); 4 Zhejiang Engineering Research Center of Intelligent Medicine, First Affiliated Hospital of Wenzhou Medical University, Wenzhou 325000, China; panzhifang@wmu.edu.cn; 5 Department of Medical Imaging and Radiological Sciences, Kaohsiung Medical University, Kaohsiung 840203, Taiwan

**Keywords:** breast cancer, tumor-infiltrating lymphocytes, molecular subtype, magnetic resonance imaging

## Abstract

**Simple Summary:**

Tumor-infiltrating lymphocytes (TILs) in breast cancer are known as promising predictive biomarkers associated with responses to neoadjuvant chemotherapy and immunotherapy, and they are also prognostic biomarkers associated with progression-free survival. The rate of high vs. low TILs in hormonal receptor 2-positive, HER2-negative (HR+/HER2−), HER2-positive (HER2+), and Triple-negative (TN) patients were compared. The high-TIL cases significantly increased from 18% in HR+ to 37% in HER2+ and 44% in TN. The MRI features among these three subtypes, as well as the features between high- vs. low-TIL cases in each subtype, were compared. The HER2+ cancer patients were more likely to present non-mass enhancement (NME). In the HR+ cohort, high-TIL cases were more likely to result in peritumoral edema. In the TN subjects, high-TIL cases were more likely to present as regular shapes and circumscribed margins. The results of this study suggest that TIL status may be determined from pre-treatment diagnostic MRI to assist in the selection of optimal treatments.

**Abstract:**

A total of 457 patients, including 241 HR+/HER2− patients, 134 HER2+ patients, and 82 TN patients, were studied. The percentage of TILs in the stroma adjacent to the tumor cells was assessed using a 10% cutoff. The low TIL percentages were 82% in the HR+ patients, 63% in the HER2+ patients, and 56% in the TN patients (*p* < 0.001). MRI features such as morphology as mass or non-mass enhancement (NME), shape, margin, internal enhancement, presence of peritumoral edema, and the DCE kinetic pattern were assessed. Tumor sizes were smaller in the HR+/HER2− group (*p* < 0.001); HER2+ was more likely to present as NME (*p* = 0.031); homogeneous enhancement was mostly seen in HR+ (*p* < 0.001); and the peritumoral edema was present in 45% HR+, 71% HER2+, and 80% TN (*p* < 0.001). In each subtype, the MR features between the high- vs. low-TIL groups were compared. In HR+/HER2−, peritumoral edema was more likely to be present in those with high TILs (70%) than in those with low TILs (40%, *p* < 0.001). In TN, those with high TILs were more likely to present a regular shape (33%) than those with low TILs (13%, *p* = 0.029) and more likely to present the circumscribed margin (19%) than those with low TILs (2%, *p* = 0.009).

## 1. Introduction

Breast cancer is the most common cancer and the leading cause of cancer-related death in women worldwide [1]. Over the past several decades, the development of emerging treatment strategies has saved the lives of many patients. Recently, novel immunotherapy has also been included for triple-negative breast cancer patients [2]. However, the choices of therapies are highly dependent on the molecular subtypes, and the prediction of responses and the prognoses is a very important aid in treatment decision making [3].

Tumor-infiltrating lymphocytes (TILs) in breast cancer are a significant component of the tumor microenvironment associated with the metabolism of tumor cells and the local immune response [4,5,6]. They have been proven as promising predictive and prognostic biomarkers. Patients with cancers of high TILs are more likely to show good responses to neoadjuvant chemotherapy and immunotherapy, particularly in human epidermal growth factor receptor 2 (HER2)-positive and triple-negative (TN) breast cancer, and have improved progression-free survival [5,7,8].

The evaluation of TILs has been a routine clinical practice in pathological examinations of breast cancer based on H&E-stained histological slides of tumor specimens [9,10]. Although the proposed assessment is elaborated in the guidelines of the International Immuno-Oncology Biomarker Working Group on Breast Cancer, the measurement of TILs still relies on visual examinations and may be highly subjective. Moreover, the distribution of TILs within the cancer is not homogeneous, and the quantitative assessment of TILs on pathological slides is limited by the sampling bias in the biopsied specimen, as well as the requirement of extensive staining in a large surgical specimen [10,11]. When the patient chooses to receive neoadjuvant chemotherapy, the current treatment regimen is very effective, and the tumor may respond very well to achieve a complete response or close to complete response. For these patients, there will be no specimen left for the histological examination of TILs in post-NAC surgery. Some studies have tried to develop fully automatic segmentation methods to provide a more comprehensive and objective assessment of TILs [12,13].

Breast MRI is known as an important imaging modality for the differential diagnosis, evaluation of disease extent, and pre-operative staging of breast cancer [14,15]. The multi-parametric breast MRI protocol can provide anatomical, vascular, and cellular information regarding the entire tumor for assessing the biological characteristics [15,16]. The morphological and functional information obtained from breast MRI has been reported to be useful in predicting molecular subtypes, response to treatment, and prognosis [17,18,19]. Some studies have shown that it is feasible to use MRI features to predict the expression level of TILs [20,21,22] and to build radiomics models to differentiate high- vs. low-TIL cases [23,24,25]. However, few studies have considered and compared TILs in different molecular subtypes.

It is known that the expression of TILs varies in different molecular subtypes which are likely associated with their differential prognoses [4,5,26,27]. Previous studies have shown that high TILs predicted a favorable prognosis in aggressive HER2+ and TN subtypes, while high TILs was a poor prognostic factor in Luminal/HER2-negative cancer [28]. Denkert et al. also reported shorter overall survival in HR+ patients with high TILs than those with low TILs [27]. Therefore, the studies analyzing the mixed subtypes were heavily dependent on the composition of subtypes, and their obtained results are not generalizable.

To further investigate the association of MRI features with TILs and subtypes, three analyses were performed in this study: (1) a comparison of the rate of high vs. low TILs in three subtypes—HR+/HER2 negative (HR+/HER2−), HER2+, and TN; (2) a comparison of the MRI features among these three subtypes; (3) a comparison of imaging features between the high- vs. low-TIL cases for each subtype.

## 2. Materials and Methods

### 2.1. Patients

This study was a retrospective study that was approved by the Institutional Review Board, and informed consent was waived. Patients with breast cancer who underwent breast MRI in our hospital from January 2017 to August 2021 were enrolled in the study. The inclusion criteria were as follows: (1) good quality of MRI for evaluation; (2) no breast surgery or biopsy, or any treatment performed before MRI examination; (3) available H&E-stained slides and pathological reports; (4) the histological type of invasive ductal carcinoma. A total of 457 patients with 457 lesions were included in the study.

### 2.2. MRI Acquisition

The GE SIGNA HDx 3.0T MR scanner with a dedicated 8-channel bilateral breast coil was used. Routine scan sequences included T2-weighted imaging (T2WI) with short-time inversion recovery (STIR); sagittal T2WI with fat suppression (fs-T2WI); T1-weighted imaging (T1WI) with dual-echo chemical-shift imaging; axial diffusion-weighted imaging (DWI) with single-shot echo planar imaging, TR 5300 ms, TE 60 ms, FA 90°, layer thickness/layer spacing 4.0/1.0 mm, FOV 34 × 34 cm^2^, matrix 128 × 128, b value 0, and 1000 s/mm^2^. Dynamic contrast-enhanced MRI (DCE-MRI) was performed after the DWI sequence using the volume imaging for breast assessment (VIBRANT) sequence, and the parameters were as follows: TR 5 ms, TE 2 ms, FA 10°, layer thickness 1.2 mm; FOV 34 × 34 cm^2^, matrix 416 × 416. The DCE series consisted of six frames: one pre-contrast and five post-contrast. The acquisition time for each frame was 90 s. The contrast agent, 0.1 mmol/kg gadopantetate dimeglumine (Magnevist; Bayer Schering Pharma, Berlin, Germany), was intravenously injected after the pre-contrast images were acquired at a rate of 2 mL/s, followed by a 20-mL saline flush at the same rate.

### 2.3. MRI Interpretation

All images were reviewed by two radiologists (with 6 and 13 years of experience, respectively) who were blind to patients’ pathological results and clinical diagnoses. Tumor characteristics were recorded as follows: largest diameter (of the largest lesion) (cm), shape (regular or irregular), margin (circumscribed or non-circumscribed), peritumor edema (present or not), lesion number (single or multiple), lesion morphology (mass or non-mass enhancement NME), ADC value, DCE kinetic pattern (wash-in/plateau/wash-out), internal enhancement pattern (homogeneous or heterogeneous). If the patient had multiple lesions, only the index lesion was evaluated. If there was a disagreement that could not be resolved by reaching a consensus, a third senior radiologist (with 25 years of experience) gave the final assessment.

Tumor size was evaluated via a multi-plane reconstruction on the second set of post-contrast images, and the maximum diameter of the lesion was measured. Peritumoral edema was defined as the presence of the hyperintensity signal around the tumor on axial or sagittal T2WI. Lesion morphology, margin, and internal enhancement patterns were analyzed on the first and second sets of the post-contrast images. ADC value was measured on the largest tumor layer that showed the most remarkable enhancement upon DCE-MRI. Three ROIs avoiding cystic, hemorrhagic, or necrosis areas were drawn, and the average ADC value was recorded.

### 2.4. Pathology

Formalin-fixed and paraffin-embedded (FFPE) tissue samples of breast cancer were used for immunohistochemistry (IHC) examinations. The status of the estrogen receptor (ER), progesterone receptor (PR), and HER2 for each cancer were determined via immunohistochemical staining. ER- or PR-positive was defined as the nuclear staining of >10% of the tumor cells. The HER2 status was considered positive when the IHC staining intensity score showed 3+. For HER2 scores of 2+, the sample was confirmed via gene amplification using fluorescence in situ hybridization (FISH). Three subtypes of breast cancer were defined: HR+/HER2−, HER2+, and TN.

**Figure 1:**
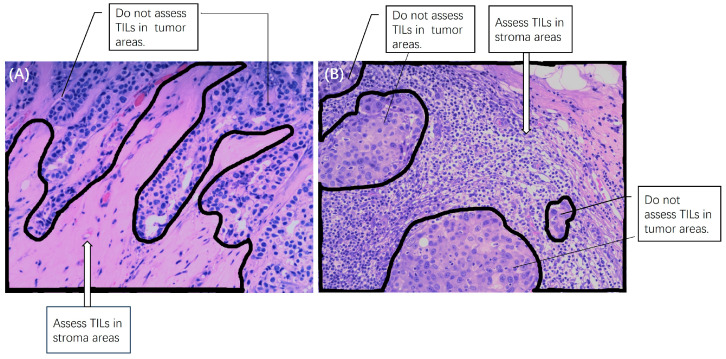
Examples of (**A**) low and (**B**) high TILs on H&E-stained slides and the assessed stroma areas with 10× magnification. TILs were assessed in the stroma areas, not inside the tumor areas.

The expression of TILs was evaluated on hematoxylin and eosin-stained (H&E-stained) slides of FFPE block with a thickness of 4.0 µm according to the recommendations outlined by the International TILs Working Group in 2014 [10]. Two pathologists with 10 and 25 years of experience carried out a consensus evaluation. The percentage of TILs in the stroma adjacent to the tumor cells was assessed. Thresholds of 10% and 50% were used as the cutoff to divide the TILs into three levels: low (<10%), medium (10–50%), and high (>50%), and because of very few >50% cases, the medium and high subgroups were combined [22,25,29,30]. Examples of low and high TILs on H&E-stained slides and assessed stroma areas are illustrated in Figure 1.

### 2.5. Statistical Analysis

Data distribution was measured using the Kolmogorov–Smirnov test or Shapiro–Wilk test. Continuous data of normal distribution are shown as mean ± standard deviation, and non-normal distribution as median (interquartile range, IQR). Categorical data are shown as frequency (percentage). In our univariate analysis, categorical variables were compared using the Chi-square test, and continuous data were compared using a one-way analysis of variance (ANOVA) or the Kruskal–Wallis test. Statistical analysis was performed using SPSS 22.0 (IBM). *p* values < 0.05 were considered statistically significant.

## 3. Results

### 3.1. Patients, Subtypes, and TILs Levels

A total of 457 patients with 457 lesions were included in the study, with 327 (71.6%) low TILs and 130 (28.4%) high TILs. The clinical and pathological characteristics of patients with low and high TILs are summarized in Table 1. The mean age of patients in the low- and high-TIL groups were 50.5 ± 9.9 and 50.7 ± 8.9 years old, respectively. Patients with high TILs presented more in ≤50 years old (*p* = 0.023) and were significantly associated with histological grade 3 and positive axillary lymph nodes (*p* < 0.001 and *p* = 0.036). Of the 241 HR+/HER2− cases, the majority 197/241 (82%) had low TILs, and only 44/241 (18%) had high TILs. There were 134 HER2+ cases, with 84/134 (63%) having low TILs and 50/134 (37%) having high TILs. Of the 82 TN, 46/82 (56%) had low TILs and 36/82 (44%) had high TILs. The composition of high TILs was significantly increased from the HR+/HER2− group to the HER2+ and TN groups (*p* < 0.001, Table 2).

### 3.2. Comparison of MRI Features among the Three Subtypes

The MRI features among the three subtypes are compared and summarized in Table 2. Eight MRI features were interpreted by radiologists. The largest diameter, lesion morphology, internal enhancement, and peritumoral edema showed significant differences among the three subtypes. The tumor size was smaller for HR+/HER2− (*p* < 0.001); HER2+ was more likely to present as NME (*p* = 0.031); homogeneous enhancement was mostly seen in HR+/HER2+ (*p* < 0.001); and the peritumoral edema was present in 45% HR+, 71% HER2+, and 80% TN (*p* < 0.001). ADC value, lesion shape, margin, and DCE kinetic pattern were not significantly different among the three subtypes.

### 3.3. Comparison of MRI Features between Cases with Low and High TILs in Each Subtype

**Figure 2:**
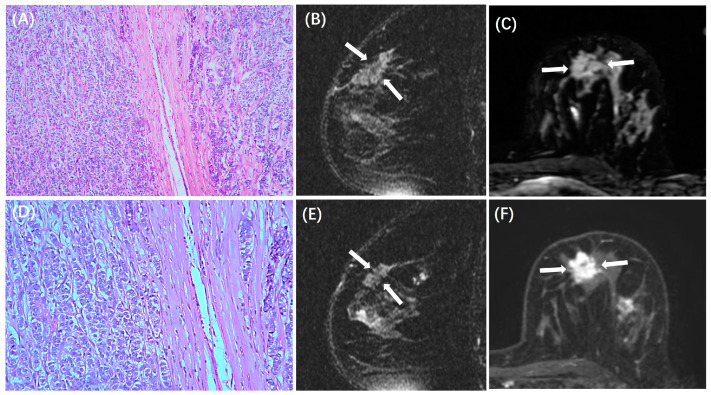
A case of HR+/HER2− breast cancer with low TILs. A 49-year-old woman with 3.0 cm IDC in the left breast. (**A**,**D**) are H&E-stained slides with 10× and 20× magnifications, respectively. (**B**,**E**) are the sagittal T2WI, and (**C**) is the axial T2WI, showing the iso-intensity signal (white arrows) of the lesion without peritumor edema. (**F**) is the axial image of the second phase of DCE-MRI, showing a prominent mass enhancement (white arrows).

**Figure 3:**
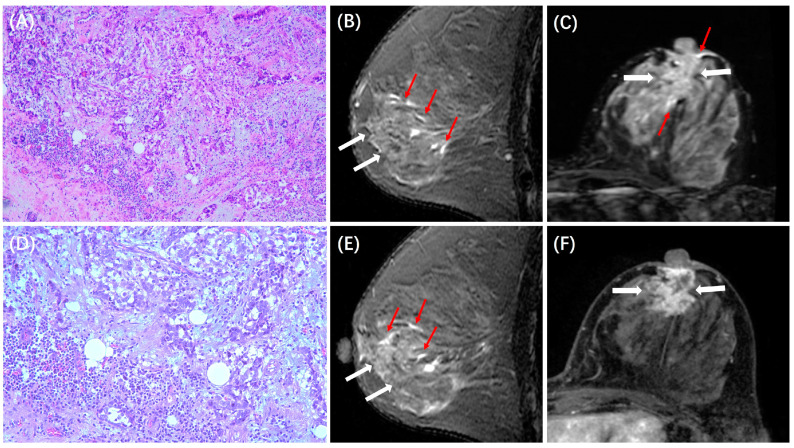
A case of HR+/HER2− breast cancer with high TILs. A 37-year-old woman with 4.0 cm IDC in the left breast. (**A**,**D**) are H&E-stained slides with 10× and 20× magnifications, respectively. (**B**,**E**) are the sagittal T2WI, and (**C**) is the axial T2WI, showing the iso-intensity signal (white arrows) of the lesion with peritumor edema (red arrows). (**F**) is the axial image of the second phase of DCE-MRI, showing a mass enhancement (white arrows).

**Figure 4:**
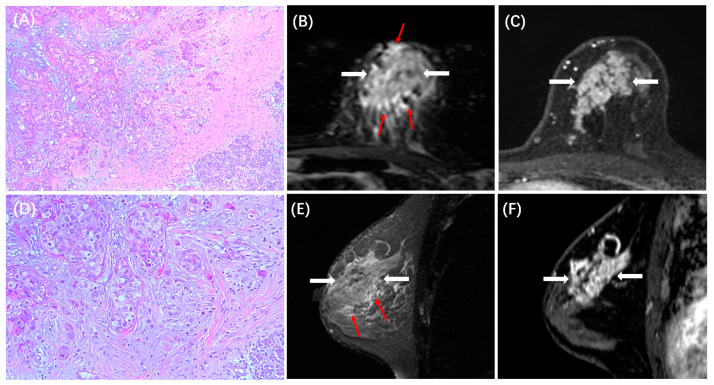
A case of HER2+ breast cancer with low TILs. A 53-year-old woman with 4.0 cm IDC in the left breast. (**A**,**D**) are H&E-stained slides with 10× and 20× magnification, respectively. (**B**,**E**) are the axial and sagittal T2WI, showing the iso-intensity signal (white arrows) of the lesion with peritumor edema (red arrows). (**C**,**F**) are the axial and sagittal images of the second phase of DCE-MRI, demonstrating non-mass enhancements (white arrows).

**Figure 5:**
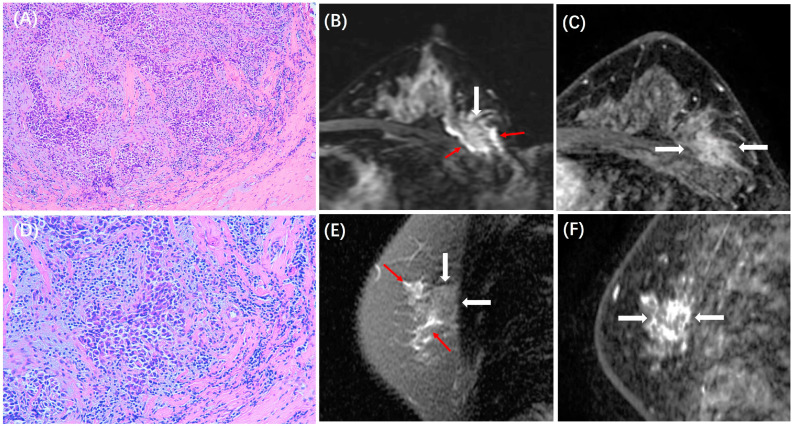
A case of HER2+ breast cancer with high TILs. A 43-year-old woman with 3.0 cm IDC in the left breast. (**A**,**D**) are H&E-stained slides with 10× and 20× magnifications, respectively. (**B**,**E**) are the axial and sagittal T2WI, showing the iso-intensity signal (white arrows) of the lesion with peritumor edema (red arrows). (**C**,**F**) are the axial and sagittal images of the second phase of DCE-MRI, demonstrating non-mass enhancements (white arrows).

**Figure 6:**
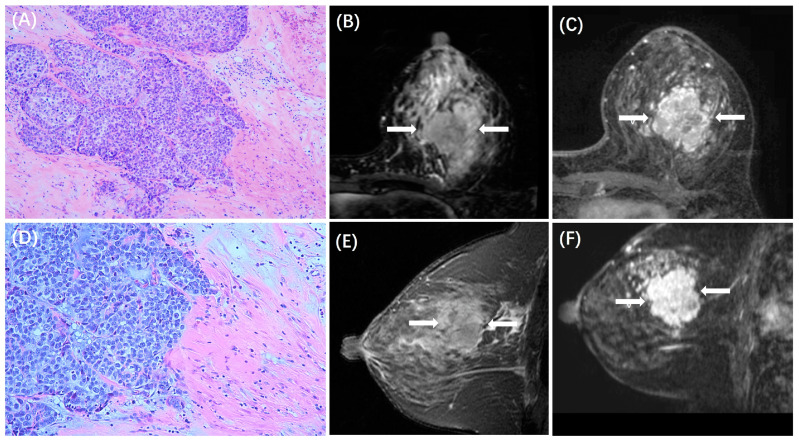
A case of TN breast cancer with low TILs. A 53-year-old woman with 4.0 cm IDC in the left breast. (**A**,**D**) are H&E-stained slides with 10× and 20× magnifications, respectively. (**B**,**E**) are the axial and sagittal T2WI, showing the iso-intensity signal (white arrows) of the lesion with irregular shapes. (**C**,**F**) are the axial and sagittal images of the second phase of DCE-MRI, showing a mass enhancement with an irregular shape and a non-circumscribed margin (white arrows).

**Figure 7:**
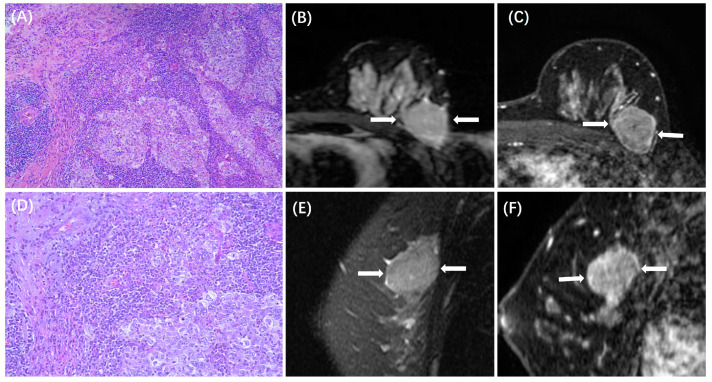
A case of TN breast cancer with high TILs. A 43-year-old woman with 3.0 cm IDC in the left breast. (**A**,**D**) are H&E-stained slides with 10× and 20× magnifications, respectively. (**B**,**E**) are the axial and sagittal T2WI, showing the iso-intensity signal (white arrows) of the lesion with regular shapes. (**C**,**F**) are the axial and sagittal images of the second phase of DCE-MRI, demonstrating a mass enhancement with a regular shape and a circumscribed margin (white arrows).

The MRI features between patients with low and high TILs in each subtype are summarized in Table 3. In the HR+/HER2− subtype, ADC value was lower in those with high TILs (0.84 ± 0.14 × 10^−3^ mm^2^/s) than in those with low TILs (0.93 ± 0.18 × 10^−3^ mm^2^/s, *p* < 0.001), and peritumoral edema was more likely to be present in those with high TILs (31/44, 70%) than in those with low TILs (78/197, 40%, *p* < 0.001). Example cases with low and high TILs of HR+/HER2− cancer are shown in Figure 2 and Figure 3, respectively. In the HER2+ subtype, there were no significant differences in MRI features between patients with low and high TILs. Example cases with low and high TILs of HER2+ cancer are shown in Figure 4 and Figure 5, respectively. In the TN subtype, those with high TILs were more likely to present a regular shape (12/36, 33%) than those with low TILs (6/46, 13%, *p* = 0.029); also, those with high TILs were more likely to present the circumscribed margin (7/36, 19%) than those with low TILs (1/46, 2%, *p* = 0.009). Example cases with low and high TILs of TN cancer are shown in Figure 6 and Figure 7, respectively.

## 4. Discussion

In this study, we first compared the rate of high vs. low TILs and the clinical and pathological characteristics of patients in in groups based on three molecular subtypes. Patients with high TILs were more likely to be younger (≤50 years old), had a higher histological grade of 3, and had positive axillary lymph nodes. The percentage of high TILs was significantly increased from the HR+ subtype to the HER2+ and TN subtypes. The MR imaging features among these three subtypes were also evaluated. The tumor size was smaller for HR+/HER2−, HER2+ was more likely to present as NME, homogeneous enhancement was mostly seen in HR+, and the peritumoral edema was mostly present in HER2+ and TN. Lastly, the features between the high- vs. low-TIL cases in each subtype were compared. In HR+/HER2−, peritumoral edema was more likely to be present in those with high TILs than in those with low TILs. In TN, those with high TILs were more likely to present a regular shape and circumscribed margin than those with low TILs.

The tumor microenvironment (TME) is known as an important factor in the growth of breast cancers and the response to chemotherapy, and TILs play a critical role in the TME [31,32]. Many studies have demonstrated the significance of TILs as prognostic or predictive biomarkers in breast cancer. However, it has been reported that the prognostic significance of TILs in breast cancer differs according to different molecular subtypes [26]. High levels of TILs were associated with a poor prognosis in ER-positive breast cancer, but it was a favorable biomarker in HER-2 and TN breast cancer [5,27,33]. When it comes to neoadjuvant chemotherapy response, a high level of TILs has a positive impact on all subtypes. The proportion of high TIL levels increases from the HR+/HER2− subtype to the HER2+ and TN subtypes as the immunogenic property increases in these subtypes [27,34]. In the present study, the rate of high TILs increased from 18% in the HR+/HER2− subtype to 37% in the HER2+ subtype and 44% in the TN subtype, respectively. In Park’s study [35], the proportion of high TILs was 45.8% in the TN subtype cohort, consistent with our results. In Denkert’s study, the proportions in the three subtypes were 45%, 56%, and 71%, much higher than ours and others [27]. The main reason for this may be that the cohort in their study comprised patients with more aggressive locally advanced tumors receiving neoadjuvant chemotherapy [30]. Low TIL levels have been observed more in early-stage surgical patients [34]. 

Molecular classification has been critical, as has histopathological information, which can be used to assess tumor biological features for the selection of the optimal treatment(s) and also for the prediction of prognoses. Breast MRI, as a noninvasive imaging modality, has a unique advantage. It is used to obtain images of the entire tumor, including surrounding tissues, and has been shown to provide information associated with molecular subtypes. Yuan et al. investigated the correlation between the imaging characteristics of DWI and DCE-MRI and the molecular subtypes and prognostic factors [35]. It was found that tumor size in the ER- and PR-positive group was smaller than the HR-negative group, and that of the HER2+ group was larger than the HER2− group. Du et al. found that a round/oval mass shape and homogeneous internal enhancement were more likely to present in Luminal A and Luminal B groups than in Luminal-HER2 and HER2-enriched groups, and the ADC value was lower among the HR+ patients than in the HR- groups and higher among the HER2+ patients than in the HER2− groups [17]. Therefore, more aggressive ER-negative breast cancers tend to be larger in size, more irregular in shape, and more heterogeneous in terms of their internal enhancement [36]. Panzironi et al. reported that peritumoral edema is associated with biologically aggressive non-luminal breast cancers characterized by large dimensions [37]. In Koh’s study, they reported that the HER2 subtype was more likely to present as NME [38]. The results of our study are consistent with these studies in the literature. The HR+/HER2− group had a smaller size than the HER2+ and TN group, and the HER2+ group showed the largest size, with a significant difference. HER2+ was more likely to present as NME than it was in the HER2− group; homogeneous enhancement was mostly seen in HR+ patients, and the presence of peritumoral edema was more present in the HER2+ and TN groups. 

Some previous studies have demonstrated that magnetic resonance imaging could be helpful in distinguishing breast cancers with low vs. intermediate or high TIL levels [23,25,39,40]. However, they did not separately analyze cases based on different molecular subtypes. In the present study, since there were significant differences in TIL expression levels and the different MRI features among the three molecular subtypes, we further evaluated the MRI features between the patients with low and high TILs in each subtype. The Luminal subtype is known to be the least immunogenic, but those with high TIL levels could also respond well to neoadjuvant chemotherapy. Therefore, the prediction of high TILs is necessary and could help select patients who would benefit from neoadjuvant therapy. Up to now, few studies have reported on the imaging features between those with high vs. low TILs based on HR+ subtype. Our results showed that, in the HR+/HER2− subtype, the ADC value was lower in those with high TILs (0.84 ± 0.14 × 10^−3^ mm^2^/s) than in those with low TILs (0.93 ± 0.18 × 10^−3^ mm^2^/s), and peritumoral edema was more likely to be present in those with high TILs (31/44, 70%) than in those with low TILs (78/197, 40%). Regarding the HER2+ subtype, there were no significant differences in MRI features between those with low and high TILs, which might be due to the small case number. Differences could be further mined using more sophisticated computational methods [22]. In a study by Lee et al., it was shown that peritumoral edema could help predict the high TILs in HER2+ breast cancer [30]. In our study, the proportion of peritumoral edema was also higher in the high TILs group in HER2+ but did not reach a significant difference. Lastly, regarding the TN subtype, those with high TILs were more likely to present a regular shape (12/36, 33%) and circumscribed margin (7/36, 19%) than those with low TILs (6/46, 13%; 1/46, 2%), which is congruent with Ku’s study [21]. 

There are several limitations to this study. First, although 457 lesions with different TIL levels meant that this study had a large series of samples compared to others published in the literature, after sorting the patients based on three molecular subtypes, the case numbers for the HER2+ and TN groups were relatively small. Second, there is no standard cutoff value available to separate the low- and high-TIL groups. We used a cutoff of 10% based on previous work [22,25,30]. Third, the data were collected in a single hospital, and only invasive ductal cancer (IDC) cases were included. Other cancer types, such as invasive lobular cancer and ductal carcinoma in situ (DCIS), are known to have different morphological presentations, and thus they were excluded to allow us to focus on the subtype differences among IDC patients. Lastly, we only presented imaging features determined from radiologists’ visual interpretations. Although the dataset can be easily used to perform a radiomics analysis and build classification models, as shown in Bian et al. [23], the clinical value of artificial intelligence (AI)-based models is being critically evaluated in the imaging field, so we opted to report findings that can be readily implemented in clinical practice.

## 5. Conclusions

In conclusion, we found that the more aggressive HER2+ and TN cancers have significantly higher TILs compared to HR+ cancers. In HR+, high-TIL cases were more likely to present peritumoral edema. In TN, high-TIL cases were more likely to present regular shapes and circumscribed margins. The MRI characteristics demonstrated an association with the low- and high-TIL groups based on the different molecular subtypes. The prediction of a high number of TILs may also assist in identifying TN patients who are likely to benefit from the inclusion of immunotherapy. 

## Figures and Tables

**Table 1:** Clinical and pathological characteristics of patients with low and high TILs.

	Low TILs (*n* = 327)	High TILs (*n* = 130)	*p*
Mean Age, years	50.5 ± 9.9	50.7 ± 8.9	0.876
Age, years			0.023
>50	177 (54%)	55 (42%)	
≤50	150 (46%)	75 (58%)	
Postmenopausal			0.249
>Yes	154 (47%)	69 (53%)	
>No	173 (53%)	61 (47%)	
Histological grade			<0.001
1	36 (11%)	1 (1%)	
2	223 (68%)	88 (68%)	
3	68 (21%)	41 (31%)	
Clinical T stage			0.061
1	202 (62%)	64 (49%)	
2	111 (34%)	60 (46%)	
3	9 (3%)	5 (4%)	
4	5 (1%)	1 (1%)	
N stage			0.036
N0	193 (59%)	68 (52%)	
N1	90 (27%)	32 (25%)	
≥N2	44 (14%)	20 (23%)	

**Table 2:** Comparison of TILs and MRI features in three molecular subtypes.

	HR+/HER2− (*n* = 241)	HER2+ (*n* = 134)	TN (*n* = 82)	*p*
TILs				<0.001
Low (<10%)	197 (82%)	84 (63%)	46 (56%)	
High (>10%)	44 (18%)	50 (37%)	36 (44%)	
MRI Tumor size (cm)	1.7 ± 1.3	2.4 ± 1.5	2.2 ± 1.2	<0.001
ADC (×10–3 mm^2^/s)	0.90 ± 0.22	0.95 ± 0.19	0.93 ± 0.19	0.091
Lesion Morphology				0.031
NME	66 (27%)	52 (39%)	20 (24%)	
Mass	175 (73%)	82 (61%)	62 (76%)	
Shape				0.247
Regular	63 (26%)	25 (19%)	18 (22%)	
Irregular	178 (74%)	109 (81%)	64 (78%)	
Margin				0.192
Circumscribed	18 (7%)	5 (4%)	8 (10%)	
Non-circumscribed	223 (93%)	129 (96%)	74 (90%)	
Internal Enhancement				<0.001
Homogeneous	35 (15%)	4 (3%)	2 (2%)	
Heterogeneous	206 (85%)	130 (97%)	80 (98%)	
DCE Kinetic Pattern				0.283
Wash-in	1 (0)	0 (0)	0 (0)	
Platform	50 (21%)	17 (13%)	13 (16%)	
Wash-out	190 (79%)	117 (87%)	69 (84%)	
Peritumoral Edema				<0.001
Present	109 (45%)	95 (71%)	66 (80%)	
Absent	132 (55%)	39 (29%)	16 (20%)	

Abbreviations: HR, hormonal receptor; HER2, human epidermal growth factor receptor 2; TN, triple-negative; NME, non-mass enhancement; DCE, dynamic contrast-enhanced.

**Table 3:** Comparison of MRI features between cases with low and high TILs in each of three molecular subtypes.

	HR+/HER2− Subtype (*n* = 241)			HER2+ Subtype (*n* = 134)			TN subtype (*n* = 82)		
	Low TILs (*n* = 197)	High TILs (*n* = 44)	*p*	Low TILs (*n* = 84)	High TILs (*n* = 50)	*p*	Low TILs (*n* = 46)	High TILs (*n* = 36)	*p*
Largest size on MRI (cm)	1.8 ± 1.3	2.1 ± 1.5	0.052	2.7 ± 2.6	2.6 ± 1.5	0.622	2.3 ± 1.1	2.4 ± 1.1	0.450
ADC (×10–3 mm^2^/s)	0.93 ± 0.18	0.84 ± 0.14	<0.001	0.94 ± 0.16	0.97 ± 0.16	0.268	0.97 ± 0.14	0.92 ± 0.16	0.095
Lesion Morphology Type			0.271			0.883			0.910
NME	51 (26%)	15 (34%)		33 (39%)	19 (38%)		11 (24%)	9 (25%)	
Mass	146 (74%)	29 (66%)		51 (61%)	31 (62%)		35 (76%)	27 (75%)	
Shape			0.571			0.128			0.029
Regular (Round/Oval)	50 (25%)	13 (30%)		19 (23%)	6 (12%)		6 (13%)	12 (33%)	
Irregular	147 (75%)	31 (70%)		65 (77%)	44 (88%)		40 (87%)	24 (67%)	
Margin			0.856			0.900			0.009
Circumscribed	15 (8%)	3 (7%)		3 (4%)	2 (4%)		1 (2%)	7 (19%)	
Non-circumscribed	182 (92%)	31 (93%)		81 (96%)	48 (96%)		45 (98%)	29 (81%)	
Internal Enhancements			0.218			0.600			0.108
Homogeneous	26 (13%)	9 (20%)		2 (2%)	2 (4%)		0 (0%)	2 (6%)	
Heterogeneous	171 (87%)	35 (80%)		82 (98%)	48 (96%)		46 (100%)	34 (94%)	
DCE Kinetic Pattern			0.601			0.854			0.859
Wash-in	1 (0)	0 (0)		0 (0%)	0 (0%)		0 (0%)	0 (0%)	
Platform	43 (22%)	7 (16%)		11 (13%)	6 (12%)		7 (15%)	6 (17%)	
Wash-out	153 (78%)	37 (84%)		73 (87%)	44 (88%)		39 (85%)	30 (83%)	
Peritumoral Edema			<0.001			0.164			0.586
Present	78 (40%)	31(70%)		56 (67%)	39 (78%)		38 (83%)	28 (78%)	
Absent	119 (60%)	13 (30%)		28 (33%)	11 (22%)		8 (17%)	8 (22%)	

Abbreviation: HR, hormonal receptor; HER2, human epidermal growth factor receptor 2; TN, triple-negative; NME, non-mass enhancement; DCE, dynamic contrast-enhanced.

## Data Availability

Data sharing is possible upon reasonable request to the corresponding author and with the approval of the institutional review board (IRB).
